# Structure elucidation of β-sitosterol with antibacterial activity from the root bark of *Malvaparviflora*

**DOI:** 10.1186/s40064-016-2894-x

**Published:** 2016-07-29

**Authors:** Mesfin Medihin Ododo, Manash Kumar Choudhury, Ahmed Hussen Dekebo

**Affiliations:** 1 Chemistry Department, Aksum University, Aksum, Ethiopia; 2 Chemistry Department, Dilla University, P.O. Box 419, Dilla, Ethiopia

**Keywords:** *Malva parviflora*, Antibacterial activity, Ethanolic extract, Chloroform extract, β-Sitosterol

## Abstract

The powder of root bark of *Malva parviflora* (Malvaceae) was successively extracted with petroleum ether (b.p. 60–80 °C), chloroform and ethanol. The chloroform extract showed antibacterial activity against *Staphylococcus aureus* and *Escherichia coli*, whereas the ethanolic extract showed antibacterial activity against only *S. aureus*. The chloroform extract, after column chromatographic separation on silica gel using petroleum ether:chloroform (3:1) as eluent, furnished 98 mg of white crystalline compound. The yield of the compound is 0.316 % (w/w). The compound has a melting point of 134–136 °C and the R_f_ value 0.56 in benzene:chloroform:acetone (1:15:1) on silica gel TLC. The compound was characterized as β-sitosterol by physical properties, chemical test, spectral analysis (FTIR, NMR and MS) and comparing the data obtained from the literature.

## Background

Populations throughout Africa, Asia and Latin America use traditional medicine to meet their primary healthcare needs (WHO 2002). Although animal parts and minerals have been used, the primary source of traditional medication is herbal medicines (WHO 2000). The majority of Ethiopians depend on herbal medicines as their only source of healthcare, especially in rural areas. Medicinal plants and knowledge of their use provide a vital contribution to human and livestock healthcare needs throughout the country areas (World Bank 2004). *Malva parviflora* is one of the most widely used herbs in Ethiopia.

**Fig. 1:**
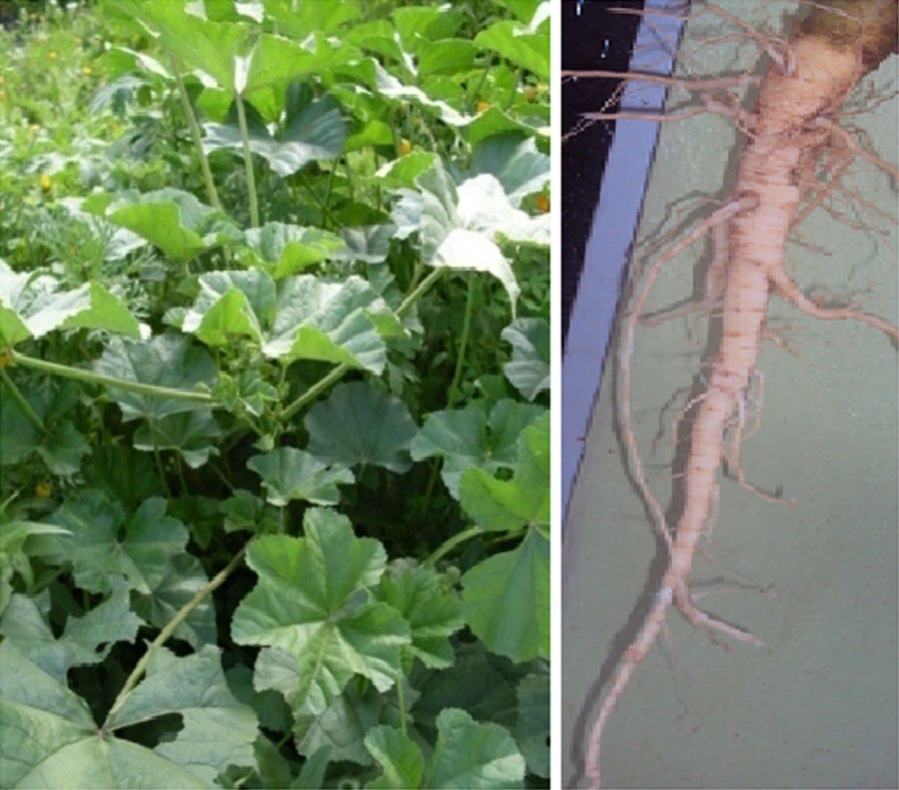
*Malva parviflora* and its root

*Malva parviflora* (family: Malvaceae) is native to Northern Africa, Europe and Asia. In Ethiopia, the plant is commonly known as *Lit* (in Amharic), *Lita* (in Afaan Oromo), *Enkefteha* (in Tigrigna) and *Uka* (in Wolaitta) languages. It often grows in waste places and agricultural farmlands. *Malva parviflora* is an annual, a biennial or a perennial herb plant. The plant is growing up to 40 in., has a deep strong tap root system and the leaves are dark green and have 5–7 toothed, rounded lobes (Fig. 1).

*Malva parviflora* has been widely used in many parts of the world for curing various diseases. In South Africa, Xhosa people use a poultice made from the whole plant parts of the plant to treat boils, inflammed purulent wounds and swellings (Afolayan et al. 2008). A hot poultice of leaf is used to treat wounds and swellings in La Reunion, and tea of the leaf is taken as a nervine tonic and used as a taenicide and for profuse menstruation (Sharma and Ali 1999). A dried powder or an infusion of the leaves and roots is used to clean wounds and sores in Lesotho (Afolayan et al. 2008). Tea of the leaf is used for treating dry, irritative cough and bronchitis (Ishtiaq et al. 2012). A decoction of the leaves and roots is also used as a hair rinse to remove dandruff and to soften the hair, and tea of the leaf is also used to clean out the mother’s system after childbirth (Mukul 2012). Seeds are demulcent, used to treat cough and ulcers in the bladder (Sharma and Ali 1999). In Ethiopia, the fresh root bark of *M. parviflora* is chopped into small pieces and applied on the *damaged* skin surfaces to treat furuncles, carbuncles, wound infections and other related ailments.

The skin infections such as furuncles and carbuncles are universally caused by *Staphylococcus aureus* (McCaig et al. 2006). The most common bacteria causing wound infection is *S. aureus* followed by *Escherichia coli* (Shittu et al. 2002; Ahmed et al. 2007). *Staphylococcus aureus* (*S. aureus*) is a Gram-positive bacterium that can live as a commensal organism on the skin and in the nose and throat (Ryu et al. 2014). Human beings are a natural reservoir for *S. aureus*; and asymptomatic colonization is far more common than infection (Chambers 2001). Approximately 30 % of healthy people are asymptomatically colonized by *S. aureus* (Ryu et al. 2014). A transmission of *S. aureus* may occur by direct contact to a colonized carrier (Chambers 2001). *Escherichia coli* is a Gram-negative bacillus that belongs to the Escherichia genus which is made up of species present in the human and other animal intestine. When eliminated in the environment together with feces, *E. coli* contaminates water, soil and food. *E. coli* strains may cause various infections including infections of the skin wounds (Moş et al. 2010).

Although *M. parviflora* plays a significant role in traditional medicine in many countries including Ethiopia, only a limited study has been done to scientifically explore antibacterial activity, and there have been no previous reports on the isolation and structural characterization of the bioactive compounds present in this plant. Thus, the present study aimed to evaluate antibacterial activity of root bark of *M. parviflora* for further structural characterization of bioactive compound(s).

## Methods

### Collection of the plant material and description the study area

Fresh plant material was collected at the agricultural farmlands area, around Shanto Town (latitude 07°01′28.2″N and longitude 037°55′09.9″E), Damot Pulassa *Woreda*, Wolaitta Zone, SNNPR, Ethiopia, in October 2012 towards the end of the rainy season. A voucher specimen (083488) has been deposited at the National Herbarium, Addis Ababa University, Addis Ababa, Ethiopia.

### Preparation of the plant material

The root parts were washed with tap water followed by distilled water. The bark part was then separated from the root and dried under shade. The dried samples were powdered using a local coffee grinder.

### Extraction

The powdered root bark of *M. parviflora* was successively extracted by Soxhlet extraction with petroleum ether (b.p. 60–80 °C), chloroform and ethanol using standard procedure as described by Jones and Kinghorn (2006). Fifteen grams of the powder was extracted in 300 mL of the solvents. The extractions were carried out for 6 h by petroleum ether, 10 h by chloroform and 15 h by ethanol. The solvents in the filtrates were then evaporated completely using water bath at their boiling points. The dried crude extracts were weighed, and the percentage (%) yields of the extractions process were calculated. The processes were repeated a total of three extractions.

### Preparation of extracts solution for antibacterial activity assay

The solutions (each 30 mg/mL) of the ethanolic and chloroform extracts were prepared by dissolving their crude extracts in sterile distilled water and dimethylsulfoxide (DMSO) respectively. The solvents were hence used as a negative control. Serial twofold dilution of each extract was prepared in a concentration ranging from 25 to 2.5 mg/mL for the determination of minimum inhibitory concentration (MIC) value. The antibacterial activity of the root bark of *M. parviflora* was compared to gentamicin (Ivee Aqua Epz Ltd, Kenya) which is a commercial synthetic antibiotic to *S. aureus* and *E. coli* pathogens (Ghalem 2014).

### Test bacteria

Two bacterial species namely *S*. *aureus* (ATCC 25923) and *E*. *coli* (ATCC 20922) were used for antibacterial activity assay. The bacteria pathogens were obtained from Ethiopian Health and Nutrition Research Institute (EHNRI), Addis Ababa, Ethiopia.

### Antibacterial activity assay

The antibacterial activity of the extracts was evaluated by Agar Well Diffusion method as described by Mattana et al. (2010) with little modification. Both bacteria strains were inoculated into 10 mL of sterile nutrient broth [Bulux Laboratories (P) Ltd, India] in respective conical flasks and incubated at 37 °C for 24 h. Nutrient agar (Merck KGaA, Germany) medium was prepared, poured into sterilized Petri dishes and allowed to solidify inside the biological safety cabinet. The cultures were then swabbed on the surface of sterile nutrient agar plates using a sterile cotton swab. Three wells (6 mm in diameter) were drilled into each plate, and the solutions of the extracts were added, until wells were filled. After in upright position incubation at 37 °C for 24 h, the diameter of inhibition zones were measured in millimeter (mm). The antibacterial activity assay was determined as the MIC value, which is the minimum concentration of the extract that could inhibit the growth of tested bacteria.

### Isolation and purification

The chloroform extract was chromatographed on silica gel 60 (0.063–0.200 mm particle size, Merck KGaA, Germany) column (11 cm length and 2 cm cross section). The silica gel was weighed using a ratio of 10 g of the adsorbent to 1 g of the extract. First, the slurry of weighed silica gel (8 g) was prepared using 100 % petroleum ether and poured into the column. The crude extract (1 g) was dissolved in 2 mL of chloroform in a beaker and adsorbed in 2 g of the silica gel. The mixture was stirred at room temperature until all the chloroform was evaporated off, and put on top of a column of previously packed. Initially, it was eluted with 100 % petroleum ether, and the polarity was gradually increased by adding chloroform at various ratios (Table 1). Totally, 118 fractions were collected (10 mL each) until the compounds were completely eluted from the column. The fractions were monitored by TLC, and then similar fractions having the same R_f_ values were combined together (Table 1).

**Table 1:** Column chromatographic separation of chloroform extract

Fractions	Solvent	Volume collected (mL)	Combined fractions	R_f_ value for major spot	Number of spots	TLC solvent
1–13	PE (100 %)	130	1–13	0.22	6	PE:CHCl_3_ (4:1)
14–29	PE:CHCl_3_ (10:1)	160	14–29	0.56	4	PE:CHCl_3_ (2:1)
30–40	PE:CHCl_3_ (9:1)	110	34–35	0.42	2	PE:CHCl_3_ (1:1)
			36–40	0.44	3	
41–48	PE:CHCl_3_ (8:1)	80	42–48	0.50	4	PE:CHCl_3_ (1:2)
49–55	PE:CHCl_3_ (7:1)	70	49–55	0.50	3	PE:CHCl_3_ (1:2)
56–62	PE:CHCl_3_ (6:1)	70	56–62	0.41	2	PE:CHCl_3_ (1:2)
63–67	PE:CHCl_3_ (5:1)	50	63–67	0.20	4	CHCl_3_ (100 %)
68–74	PE:CHCl_3_ (4:1)	70	68–74	0.20	4	CHCl_3_ (100 %)
75–89	*PE:CHCl*_*3*_ *(3:1)*	150	75–77	0.55	4	*CHCl*_*3*_ *:Act (15:1)*
			*80*–*89*	*0.50*	*3*	
90–101	PE:CHCl_3_ (2:1)	120	90–101	0.21	3	CHCl_3_:Act (9:1)
102–133	PE:CHCl_3_ (1:1)	320	102–110	0.46	2	CHCl_3_:Act (10:1)
			111–118	0.44	3	

Italics characters are used for the conditions that the compound has been isolated

*PE* petroleum ether, *CHCl*_*3*_ chloroform, *Act* acetone

Fractions (80–89), showed a major spot with two faint spots, were kept in a fridge at 4 °C for 4 h after addition of a little methanol. The crystals were formed at the bottom of the flask and separated from the mother liquor. The light yellow crystals were further purified by crystallization from methanol (white crystal, 98 mg). The yield of the compound is 0.316 % (w/w). The purity of isolated compound was determined by TLC and melting point before submitting to the spectral analysis.

### Thin layer chromatography (TLC)

A small amount of crystals was dissolved in chloroform and spotted on the TLC plate using pre-coated aluminium with 0.20 mm thick silica gel 60 F_254_ (20 × 20 cm, Merck KGaA, Germany). Then the TLC plate was run by the solvent system of benzene:chloroform:acetone (1:15:1), respectively. After drying at room temperature, the spot was visualized by placing the plate in iodine vapor. The retardation factor (R_f_) value was then measured.

### Melting point measurement

A melting point (m.p.) measurement was performed by procedure as described by Brittain (2009) on a SA-300H digital melting point apparatus. A few crystals of the compound were placed in a thin walled glass capillary tube and then inserted into the side of the heating block via the hole provided in the apparatus. The compound was then heated until it was melted. The temperature at which the solid began to melt, and that at which it was completely liquid, was recorded as the melting point range of the compound.

### Spectra measurement

FTIR spectrum was recorded on a Perkin Elmer 1330 spectrometer with KBr pellets. ^1^H and ^13^C NMR spectra were measured on a Bruker DPX 400 MHz and 100.06 MHz spectrometer respectively. High-resolution electrospray ionization mass spectrometry (HR–ESI–MS) measurement was carried out on a Q-Tof Micro YA263 spectrometer in the positive ion mode.

### Test for steroid

The test for steroid was performed by Liebermann–Burchard reaction as described by Rajput and Rajput (2012). A few crystals of isolated solid compound were dissolved in chloroform, and a few drops of concentrated sulfuric acid were added to the solution followed by the addition of 3 drops of acetic anhydride. The solution turned violet blue and finally green.

### Statistical analysis

The extraction efficiency of the solvents and the zones of inhibition induced by the plant extracts against tested bacteria were given as mean ± SD (where SD is standard deviation) of three replicates. Difference between means zones of inhibition and the standard was determined using students’ test (t-test). The level of statistical significance was set at *P* ≤ 0.05.

## Results and discussion

### Extraction

Petroleum ether gave light yellow coloured solid (1.18 ± 0.07 %), chloroform yielded light brown coloured solid (3.22 ± 0.42 %) and ethanol resulted yellow coloured amorphous solid (9.62 ± 0.84 %) crude extracts. The results revealed that different solvents have been found to extract different active principles depending on their polarity, and ethanol could extract the highest amount of materials present in the plant which indicates that the root bark of *M. parviflora* contains the largest proportion of polar components. The percent yield of petroleum ether is very low and could not allow further chemical study. In the present study, we conducted only the antibacterial activity evaluation on chloroform and ethanolic crude extracts.

### Antibacterial activity assay

**Fig. 2:**
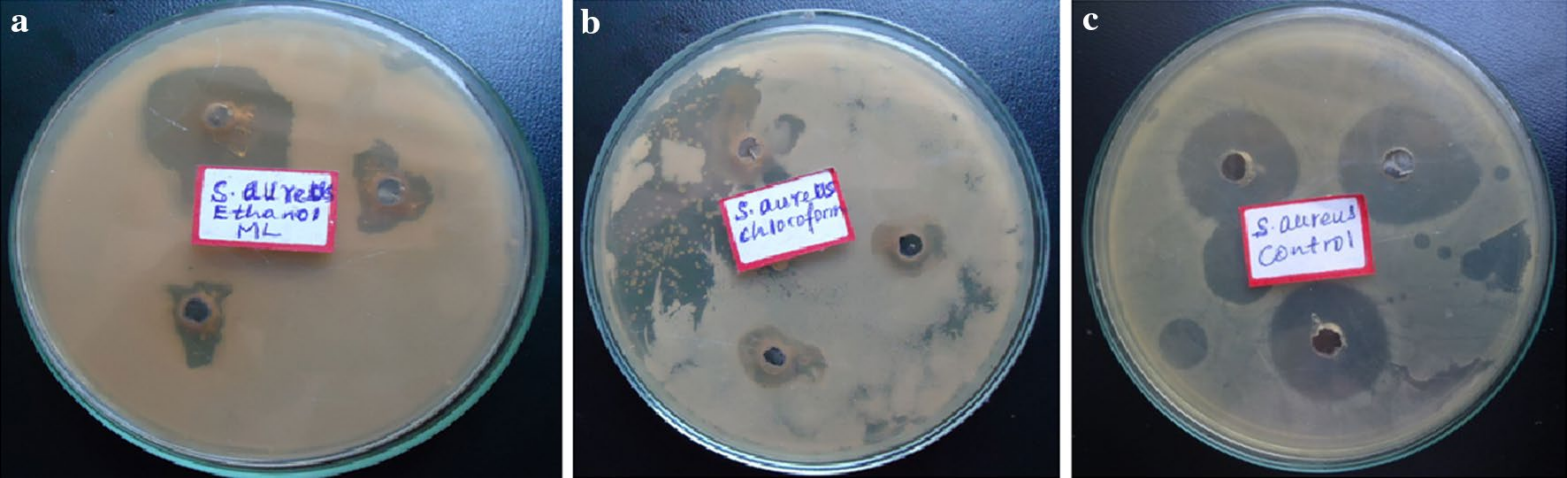
Growth inhibition of *S. aureus* by **a** ethanolic extract, **b** chloroform extract, **c** gentamicin

**Fig. 3:**
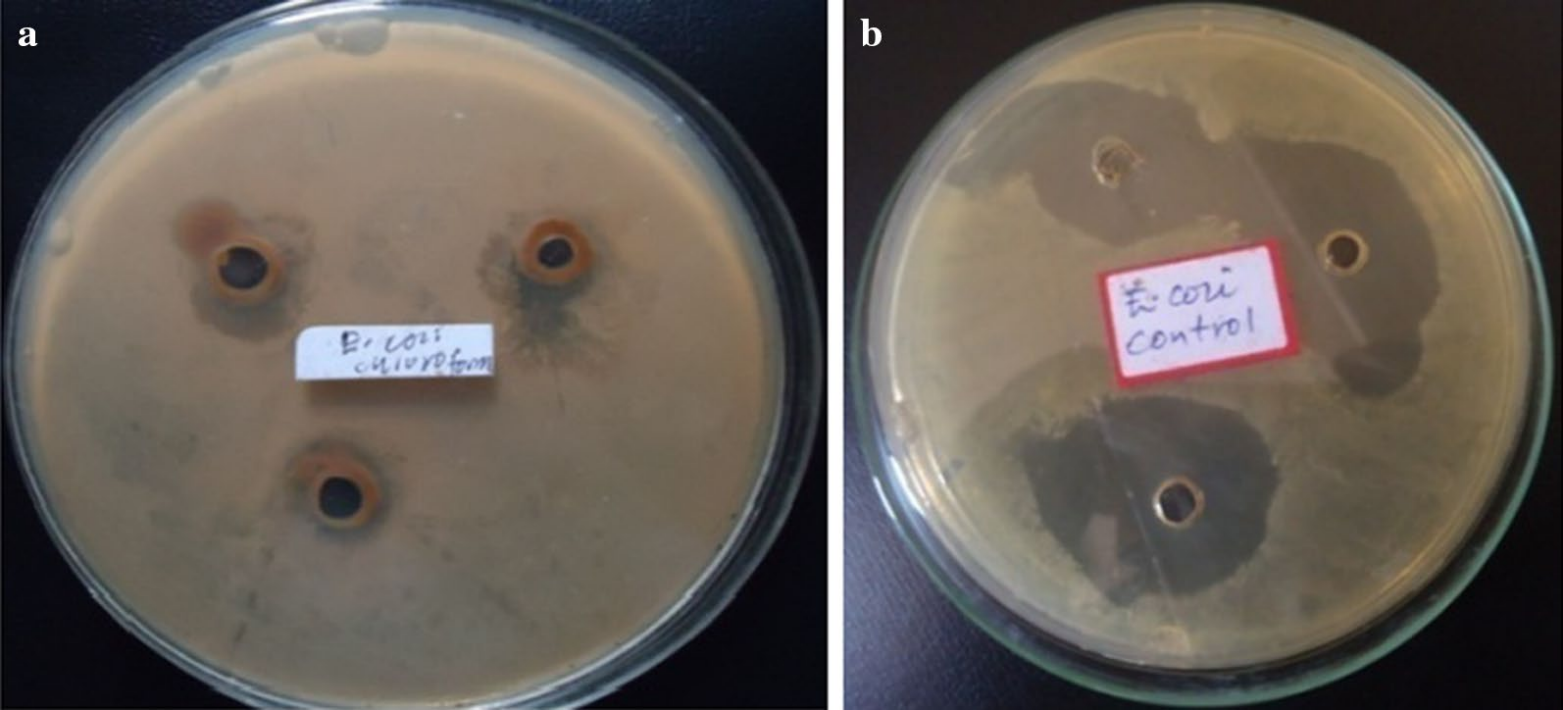
Growth inhibition of *E. coli* by **a** chloroform extract, **b** gentamicin

The chloroform extract showed antibacterial activity against *S. aureus* (Fig. 2b) and *E. coli* (Fig. 3a) with the same diameter of zones of inhibition (15 ± 0.41 mm) and MIC value of 20 mg/mL, whereas the ethanolic extract showed antibacterial activity against only *S. aureus* (Fig. 2a) with diameter of zone of inhibition (18 ± 3.20 mm) and MIC value of 15 mg/mL. The results revealed that *S. aureus* more sensitive to ethanolic extract than chloroform extract. This is due to stronger extraction capacity of ethanol could have produced greater number of active constituents responsible for antibacterial activity against *S. aureus*. However, only chloroform extract showed antibacterial activity against *E. coli*. The fact that the root bark of *M. parviflora* showed antibacterial activity against *S*. *aureus* and *E. coli* might justify the use of this plant in traditional medicine for the treatment of the diseases caused by tested bacteria pathogens.

**Fig. 4:**
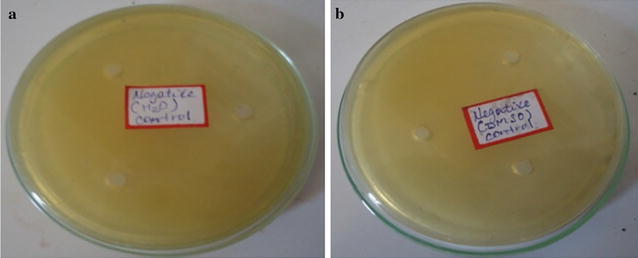
No growth inhibitions of *S. aureus* and *E. coli* by **a** sterile distilled water, **b** DMSO

It was observed that *S. aureus* (Gram positive bacterium) is highly susceptible to inhibition by ethanolic extract, whereas *E. coli* (Gram negative bacterium) did not show growth of inhibition. This may probably be due the morphological differences particularly cell wall composition between the two bacteria species. The Gram-positive bacteria have only an outer peptidoglycan layer which is not effective permeability barrier, whereas the most of the Gram-negative bacteria possess an outer multilayered peptidoglycan and a phospholipidic bilayer. This makes the cell wall impermeable to most of the drugs (Ghansar et al. 2012). Gentamicin (a positive control) caused zones of inhibition 24 ± 1.21 mm (Fig. 2c) and 25 ± 1.04 mm (Fig. 3b) against *S. aureus* and *E. coli* respectively. The statistical evaluation revealed that the root bark of *M. parviflora* showed significantly lower inhibitory activity against both tested bacteria than the standard antibiotic. Sterile distilled water (Fig. 4a) and DMSO (Fig. 4b) did not show any antibacterial activity against tested bacteria.

There have been a few previous reports on the antibacterial activity of *M. parviflora*. The root of *M. parviflora* inhibited the growth of *S. aureus* and *E. coli* with the zones of inhibition ranged between 0.20 and 0.43 mm (Shale et al. 2005). According to Tadeg et al. (2005) report, the root of *M. parviflora* showed zone of inhibition (20 ± 0.0 mm) against *S. aureus*, but no zone of inhibition was noted against *E. coli*. Furthermore, Kalayou et al. (2012) performed antibacterial activity on the leaves of *M. parviflora*, and the zones of inhibition were 9.70 ± 1.10 mm for *S. aureus* and 10.25 ± 2.20 mm for *E. coli*. In general, the zones of inhibition obtained in the present study are different from results in the previous reports. This is due to several variables which influence the bioactive plant constituents against tested bacteria such as the environmental and climatic conditions under which the plant grow, choice of plant extracts, choice of extraction methods and antimicrobial test method as well (Ncube et al. 2008).

### TLC, melting point and steroid test

The isolated compound showed a single spot with R_f_ value 0.56 and has m.p. of 134–136 °C. A sharp m.p. (just a narrow range of 1–2 °C) indicates that the high purity of the isolated compound (Brittain 2009). The m.p. result of the compound is very close to the literature value (134–135 °C) for β-sitosterol (Hang and Dussault 2010). In steroid test, the compound showed a violet-blue colour, which finally turned into green in Liebermann–Burchard reaction indicating the presence of steroid (Rajput and Rajput 2012).

### Spectra analysis

**Fig. 5:**
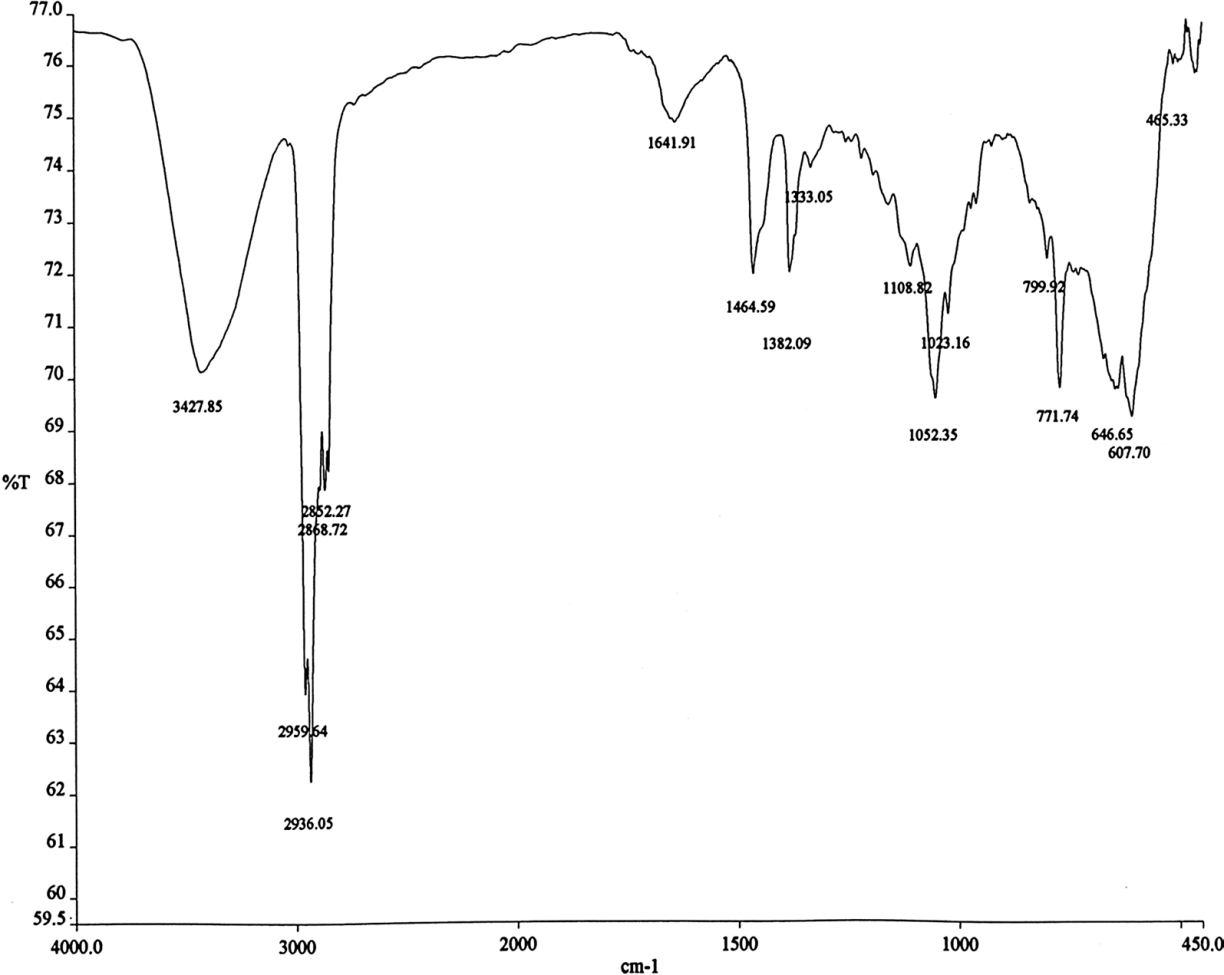
The FTIR spectrum

The FTIR spectrum (Fig. 5) showed absorptions band (cm^−1^) for OH at 3427.85, CH_3_ at 2959.64 and 2868.72, CH_2_ at 2868.72 and 2852.27, unconjugated olefinic (C=C) at 1641.91, cyclic methylene groups (CH_2_)n at 1464.59, gem-dimethyl (–CH(CH_3_)_2_) group at 1382.09 and C–OH of secondary alcohol at 1052.35.

**Fig. 6:**
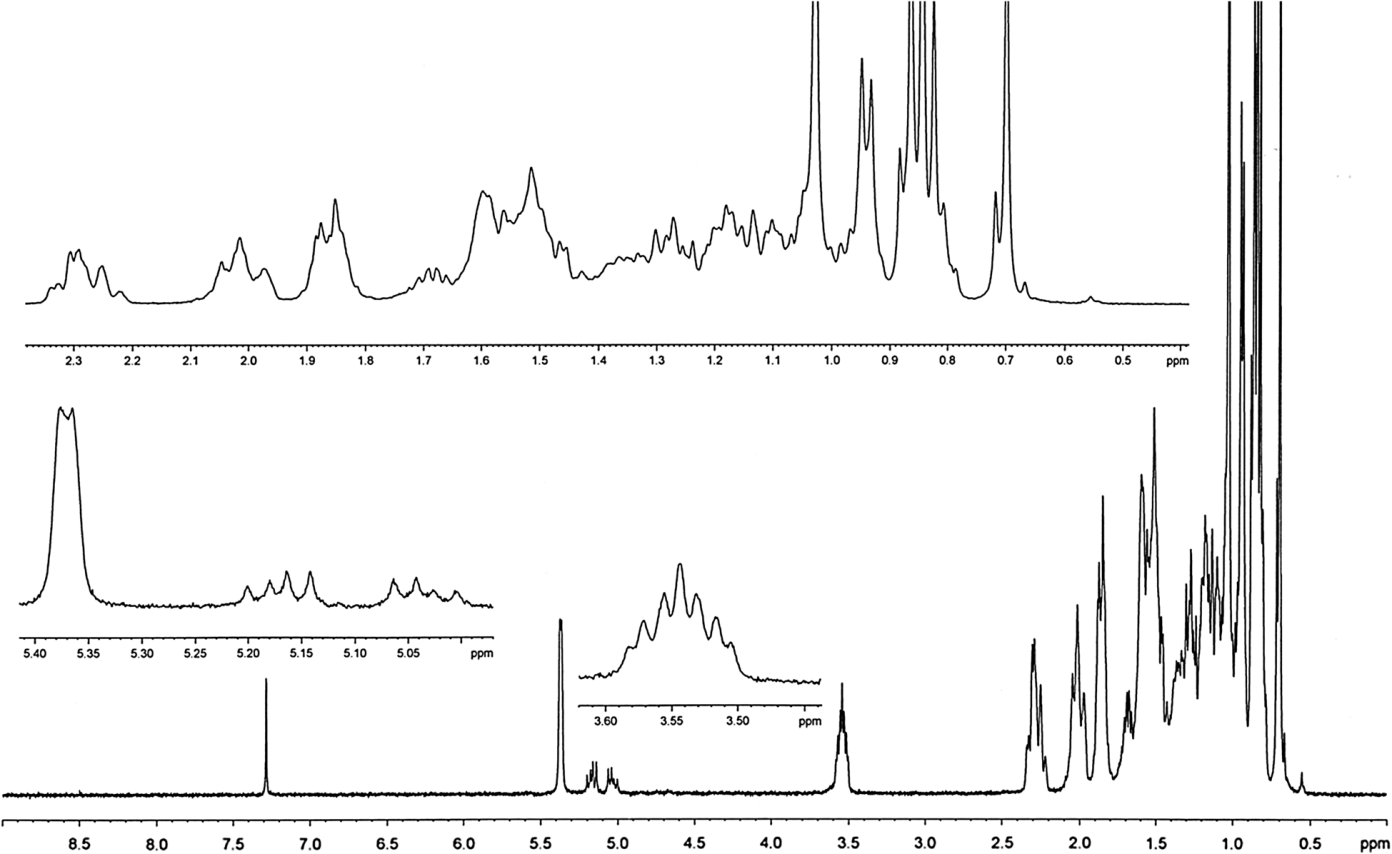
The ^1^H NMR spectrum

The integration of ^1^H NMR spectrum (Fig. 6) showed the presence of fifty hydrogens: six *CH*_*3*_, eleven *CH*_*2*_, nine *CH* and one *OH* groups (Table 2). The appearance the singlets at δ 0.70 and 1.03 confirming the presence of two *CH*_*3*_ attached to quaternary carbons. The appearance of the complex multiplets at δ 2.29 and 2.32 is revealed that the two *CH*_*2*_ adjacent to carbon attached to *OH* group. The multiplet at δ 3.54 is due to a proton connected to the carbon which attached with *OH* group. The overlapping triplet signal also appeared for *CH* at δ 5.37 indicated that the presence of one olefinic proton.

**Table 2:** The observed ^1^H and ^13^C NMR spectra data in CDCl_3_ with a drop of methanol-*d*_4_ at 400 and 100.06 MHz, respectively

Position	Type	Chemical Shift, δ (ppm) value	
		^13^C NMR	^1^H NMR (multiplicity)
1	CH_2_	37.28	1.46 (m)
2	CH_2_	31.69	1.56 (m)
3	CH(OH)	71.82	3.54 (m)
4	CH_2_	42.33	2.32 (m)
5	QC(=)	140.77	–
6	CH(=)	121.73	5.37 (overlapping, t)
7	CH_2_	31.93	2.04 (m)
8	CH	31.93	1.69 (m)
9	CH	50.16	1.55 (m)
10	QC	36.51	–
11	CH_2_	21.11	1.52 (m)
12	CH_2_	39.80	1.51 (m)
13	QC	42.34	–
14	CH	56.79	1.50 (m)
15	CH_2_	24.33	1.58 (m)
16	CH_2_	28.27	1.85 (m)
17	CH	56.08	1.45 (m)
18	CH_3_	11.89	0.70 (s)
19	CH_3_	19.42	1.03 (s)
20	CH	36.17	1.60 (m)
21	CH_3_	18.84	0.94 (overlapping, d)
22	CH_2_	33.98	0.93 (m)
23	CH_2_	26.11	1.15 (m)
24	CH	45.86	1.38 (m)
25	CH	29.19	1.57 (m)
26	CH_3_	19.84	0.84 (overlapping, d)
27	CH_3_	19.06	0.86 (d)
28	CH_2_	23.10	1.10 (m)
29	CH_3_	12.01	0.82 (overlapping, t)
**–**	OH	–	1.98 (s)

**Fig. 7:**
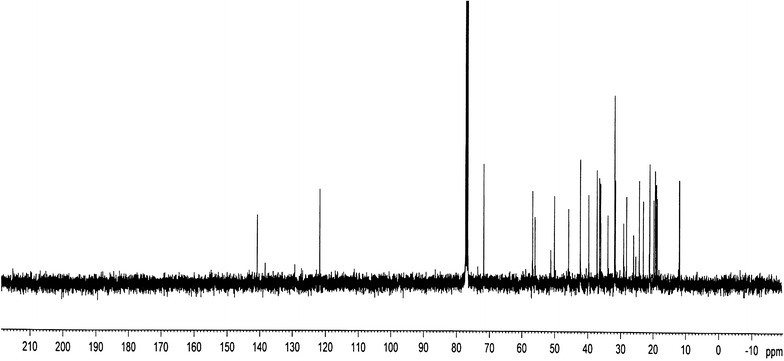
The ^13^C NMR spectrum

**Fig. 8:**
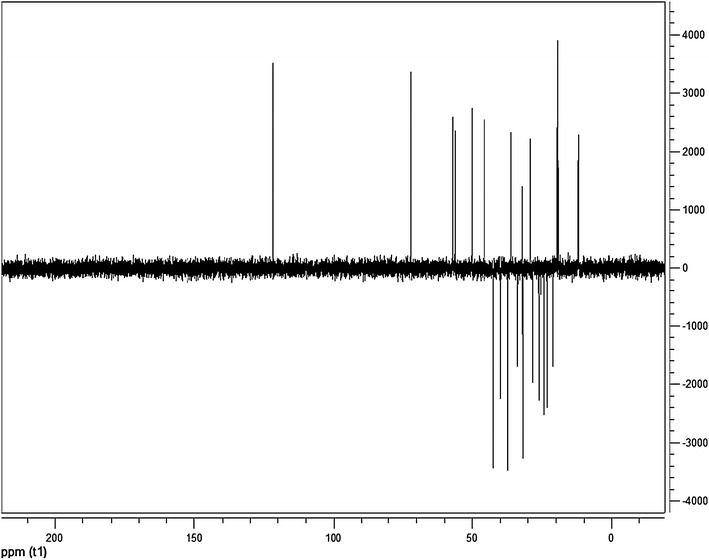
The DEPT-135 spectrum

The ^13^C NMR spectrum (Fig. 7) exhibited the existence of 29 carbons. The carbons could be classified as representing *CH*_*3*_, *CH*_*2*_, *CH* or quaternary carbon (QC) by DEPT-135. The DEPT-135 spectrum (Fig. 8) indicated the presence of 26 carbons: six peaks appeared up due *CH*_*3*_ groups, nine peaks up for *CH* groups and peaks appeared down indicated the presence of eleven *CH*_*2*_ groups (Table 2). The absence of three signals in the DEPT-135 spectrum confirmed the presence of three QC atoms. In ^13^C NMR spectrum, the recognizable signals at 140.77 and 121.73 are assigned for double bond between carbon atoms in position 5 and 6 (C_5_ = C_6_), respectively. The signal at δ 71.8 is assigned for C_3_ β-OH group, and the signals at δ 11.89 and 19.42 are assigned for angular methyl carbons for C_19_ and C_18_, respectively. The chemical shift value for C_18_ is lower due to γ-gauche interaction that increases the screening of the C_18_. However, the loss of H-atom in C_6_ results in decrease in screening of the C_19_ leading to increase in ^13^C NMR chemicals shift to higher frequency (Pateh et al. 2009).

**Fig. 9:**
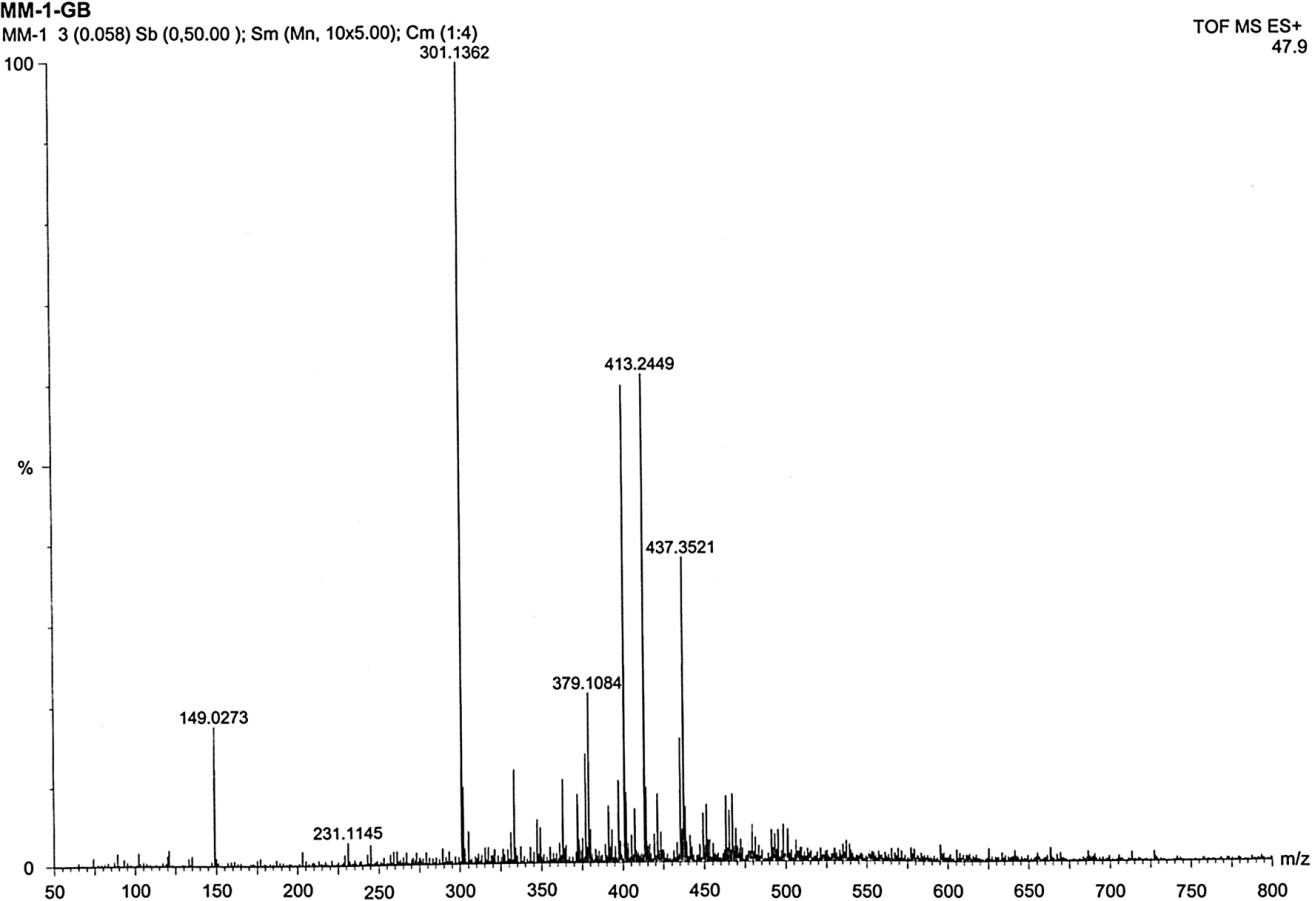
The HR–ESI–MS spectrum

The mass molecular ion of the compound appeared in HR–ESI–MS spectrum (Fig. 9) at m/z 437.3521 which is approximately 23 higher than the expected because the compound was ionized under positive mode HR–ESI by addition of Na atom. This indicated that the isolated compound with molecular weight of 414.3521, in good agreement with the theoretical value (calculated for C_29_H_50_O, 414.7066). The characteristic peak was given at m/z 413.2449 that corresponds to (M‐1) or loss of H. The spectrum showed the most intense peak at m/z 301.1362 that corresponds to (M‐113) or loss of (–C_8_H_17_). Other ion peak at m/z 231.1145 is due to the loss of side-chain and ring D fragment, –C_13_H_27_ that corresponds to the M-183.

The observed chemical shift values (Table 2) in NMR spectra are very close to values reported in the literature for β-sitosterol (Patra et al. 2010).

**Fig. 10:**
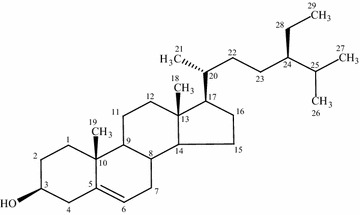
The structure of β-sitosterol

Based on ^1^H NMR, ^13^C NMR, DEPT-135 and HR–ESI–MS data, molecular formula of the isolated compound was determined to be C_29_H_50_O. Since the isolated compound gave positive test for steroids, all of the other structures other than steroids were rejected. Based upon the functional group analysis, it was found that the nature of oxygen was hydroxyl which is supported by FTIR data. The FTIR spectrum also showed the presence of one C=C in the structure. So, the steroids with other functional groups were rejected. In general, based on the physical properties (crystal with white color and m.p.), steroid test and spectroscopic data (IR, NMR and MS) and comparing the data in the scientific literature, the structure of the isolated compound was determined to be β-sitosterol (Fig. 10).

β-Sitosterol is a natural micronutrient found in the cells and membranes of all oil producing plants, fruit, vegetables, grains, seeds and trees (Sen et al. 2012). It is commercially available in preparative amounts only as mixtures with other phytosterols typically stigmasterol and campesterol (Hang and Dussault 2010). β-Sitosterol has been proven to be a safe, nontoxic, effective nutritional supplement and has amazing potential health benefits in many diverse applications including antibacterial activity (Sen et al. 2012). Earlier experimental studies have shown that β-sitosterol has antibacterial activity against different bacteria species including *S*. *aureus* and *E*. *coli*. According to Sen et al. (2012) and Joy et al. (2012) reports, β-sitosterol inhibited the growth of *S*. *aureus* (17.83 ± 0.58 mm) and *E. coli* (14.5 ± 1.84 mm) and *S*. *aureus* (13 mm) and *E. coli* (14 mm) respectively. So, the study suggested that the presence of β-sitosterol in chloroform extract of the root bark *M. parviflora* might contribute to its potency of growth inhibition against tested bacteria.

## Conclusions

The antibacterial activity assay showed that the ethanolic and chloroform extracts of the root bark *M. parviflora* possess active compound to inhibit the growth of bacteria species: *S. aureus* and *E. coli*. This is in agreement with the use of this plant in traditional medicine for the treatment of furuncles, carbuncles, wound infections and other related ailments. In chromatographic separation, β-sitosterol was isolated from chloroform extract of the root bark of *M. parviflora*. Although β-sitosterol is a known natural product, this is the first report of the isolation from this plant and its structural characterization.
